# An Overview of Physical, Microbiological and Immune Barriers of Oral Mucosa

**DOI:** 10.3390/ijms22157821

**Published:** 2021-07-22

**Authors:** Sevda Şenel

**Affiliations:** Department of Pharmaceutical Technology, Faculty of Pharmacy, Hacettepe University, Ankara 06100, Turkey; ssenel@hacettepe.edu.tr

**Keywords:** oral epithelium, cell junctions, oral immunity, oral microbiome, epithelial barrier, personalized medicine, antimicrobial peptides, permeability

## Abstract

The oral mucosa, which is the lining tissue of the oral cavity, is a gateway to the body and it offers first-line protection against potential pathogens, exogenous chemicals, airborne allergens, etc. by means of its physical and microbiological-immune barrier functions. For this reason, oral mucosa is considered as a mirror to the health of the individual as well as a guard or early warning system. It is organized in two main components: a physical barrier, which consists of stratified epithelial cells and cell–cell junctions, and a microbiological-immune barrier that keeps the internal environment in a condition of homeostasis. Different factors, including microorganism, saliva, proteins and immune components, have been considered to play a critical role in disruption of oral epithelial barrier. Altered mucosal structure and barrier functions results in oral pathologies as well as systemic diseases. About 700 kinds of microorganisms exist in the human mouth, constituting the oral microbiota, which plays a significant role on the induction, training and function of the host immune system. The immune system maintains the symbiotic relationship of the host with this microbiota. Crosstalk between the oral microbiota and immune system includes various interactions in homeostasis and disease. In this review, after reviewing briefly the physical barriers of oral mucosa, the fundamentals of oral microbiome and oral mucosal immunity in regard to their barrier properties will be addressed. Furthermore, their importance in development of new diagnostic, prophylactic and therapeutic strategies for certain diseases as well as in the application for personalized medicine will be discussed.

## 1. Introduction

The mucosa, which consists of epithelial cells, acts as a barrier by forming a continuous layer and protects the body from environmental exposures, physical and chemical damage, microbes and toxins through its physical and immunological barrier functions. According to the needs of these tissues, the structure and the function of the epithelial cells differs between the skin, gastrointestinal system and the respiratory tract, which are the main interfaces between the host and the environment. For example, oral mucosa protects the deeper tissues from mechanical insults, and also prevents the entry of bacteria and some toxic substances into the body, while skin provides a strong physical barrier; respiratory epithelial cells provide continuous particle clearance for a healthy gas exchange in the lungs and intestinal epithelial cells provide extensive nutrient and water exchange. In this review, the focus will be the oral mucosa, which is the first-line protection barrier of the body, consisting of physical and microbiological-immune barriers. Understanding the function of oral mucosal barriers is essential for a wide variety of research areas such as cancer, inflammation and infection diseases, dentistry, drug formulation and biomarkers. The functionality of the barrier is regulated by its microenvironment and often altered during pathological conditions or other external factors. Since it is a very broad subject, herein it is aimed to give a general overview of the oral mucosal barriers, and the reader will be directed to the comprehensive review and research articles for the details on the related areas. 

## 2. Oral Epithelium and Cell Junctions

**Figure 1:**
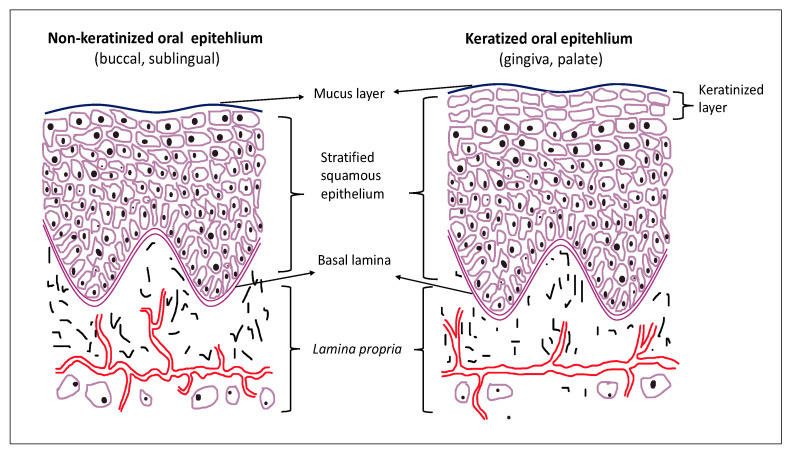
Structure of the oral mucosa.

The oral mucosa lining the oral cavity functions to protect the underlying tissue from mechanical damage, besides functioning as a primary barrier site and a portal for the entry of food, microbes and airborne particles into the gastrointestinal tract. It comprises stratified squamous epithelium and an underlying connective tissue termed *lamina propria* (Figure 1). The epithelial surface is kept moist with mucus produced by the major and numerous minor salivary glands. In different regions of the oral cavity, the mucosa shows adaptation to differing mechanical demands. Masticatory mucosa (hard palate and gingiva) consists of a keratinized epithelium tightly attached to the underlying tissues by a collagenous connective tissue, whereas lining (buccal, sublingual) mucosa comprises a nonkeratinized epithelium supported by a more elastic and flexible connective tissue [1]. The epithelium is constantly replaced by cell division in the deeper layers, and turnover is faster in the lining than in the masticatory regions. The epithelium on the dorsum of the tongue is a specialized epithelium, which is considered as a mosaic of keratinized and nonkeratinized epithelia. 

**Figure 2:**
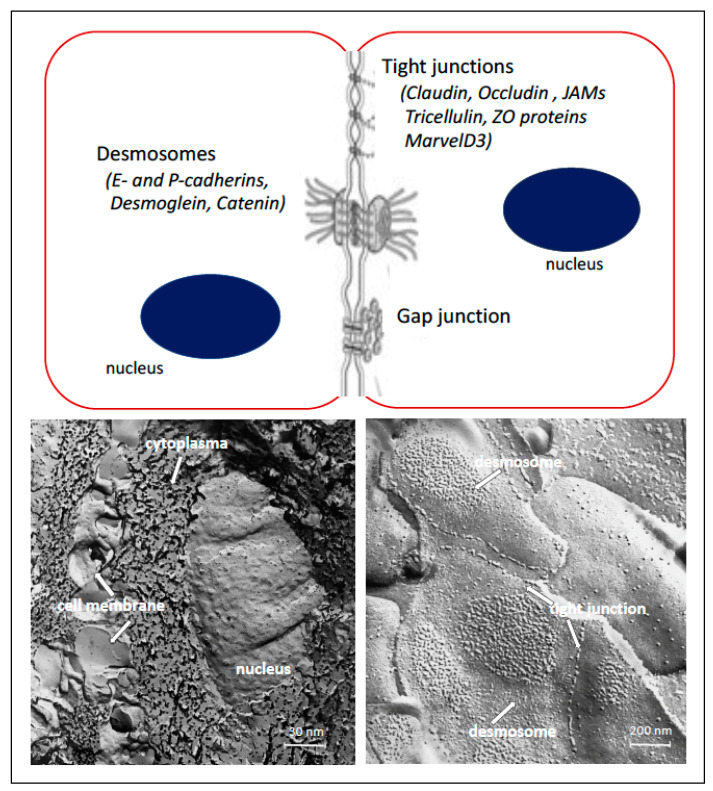
Cell junctions in the oral epithelium and freeze fracture micrographs of intact buccal epithelium; freeze fracture micrograph on the left side depicts cell nucleus, cytoplasm and cell membrane, on the right side, TJs and desmosomes between cells are shown at larger magnification.

Epithelium is separated and bound with strong integrin bonds to the underlying extracellular matrix tissue by a fibrous basement membrane. Physical barriers are created by the unique architectural integrity of the stratified epithelia, where epithelial cells are interconnected by tight junctions (TJs) (occluding junction), gap junction and anchoring junctions (desmosomes and adherens junctions) (Figure 2) [2,3]. In this way, oral epithelium provides the first line of defense against a diverse range of environmental and microbial irritants. Cell–cell interactions are essential in many physiological processes of the epithelium, and they can be rapidly rearranged in themselves under different physiological and pathological conditions. Yet, in presence of some diseases, such as infectious diseases, autoimmune diseases and cancer, they can be deregulated. 

Cell production in the deeper layers of the epithelium is balanced by the loss of cells from the surface. There is a rapid clearance of surface cells, which acts as a protective mechanism by limiting colonization and invasion of the microbes adherent to the mucosal surface. Various external factors as well as chemotherapeutic agents and radiation may limit proliferation of the epithelium so that it becomes thin or ulcerated, mainly in the lining regions. 

In the stratified oral epithelium, TJs are present in the superficial layers and are known to be the main barrier function of the epithelium. TJs are complex protein structures that seal the adjacent epithelial cells together. They prevent the passage of most dissolved molecules, microbes and toxins from one side of the epithelial layer to the other, thereby organizing number of different signaling and trafficking molecules, which regulate cell differentiation, proliferation and polarity [4]. Among the transmembrane proteins which forms the TJs are occludin, claudin, tricellulin and MarvelD3 (which all belong to the TJ-associated MARVEL domain containing protein (TAMP) family) junctional adhesion molecules (JAMs) [3,5,6,7,8,9]. In addition, there are peripheral intracellular membrane proteins that connect transmembrane TJ molecules and actin filaments such as the zonula occludens (ZO) proteins. Claudin and occludin, which are the main TJ proteins, have four transmembrane domains and two extracellular loops, while JAMs have immunoglobulin-like domains. Due to the altered expression of these proteins, TJs have been linked to various diseases that affect many tissues and organs. Some of these diseases are inherited, and involve mutations or polymorphisms of the TJ proteins or may cause activation of TJ-associated signaling mechanisms. Many pathogenic viruses and bacteria can also target TJs interacting with the junctional proteins [7]. Mislocalization of the tight junction proteins, occludin and claudin in epithelial layers was reported to result in apoptosis through the extrinsic pathway as well [10]. Although many diseases, such as chronic inflammatory conditions and cancer, have been linked to dysfunction of the tight junctions, it is not always possible to know whether this is the cause or the result of the disease [11]. 

The adherens junctions and desmosomes, which are localized below tight junctions, maintain cell–cell adhesion. Both anchoring junctions are linked to the cytoskeletal filaments and provide scaffolds for the maintenance of tissue integrity. They are important for maintaining tissue architecture and cell polarity, and can limit cell movement and proliferation [12]. The major component of the adherens junctions is the transmembrane protein E-cadherin. Cadherins associate with cytoplasmic proteins, called catenins, which in turn bind to cytoskeletal components, such as actin filaments and microtubules. The desmosomal cadherin subfamily consists of desmogleins [13,14]. The adhesion cores of the junctions consist of the proteins that mediate direct interactions between adjacent cells, while on the cytoplasmic site the proteins are coupled to cytoskeleton via a collection of adaptor proteins [2]. Gap junctions are clusters of intercellular channels facilitating a direct connection between the cytoplasm of two neighboring cells to mediate intercellular communication. These channels are formed connexons, which are oligomerized from connexin proteins (Cxs) [2]. Cxs and gap junctions have been reported to play important roles in maintaining the normal development and function of oral tissues [15]. 

## 3. Permeation Barrier of Oral Epithelium to Drugs

**Figure 3:**
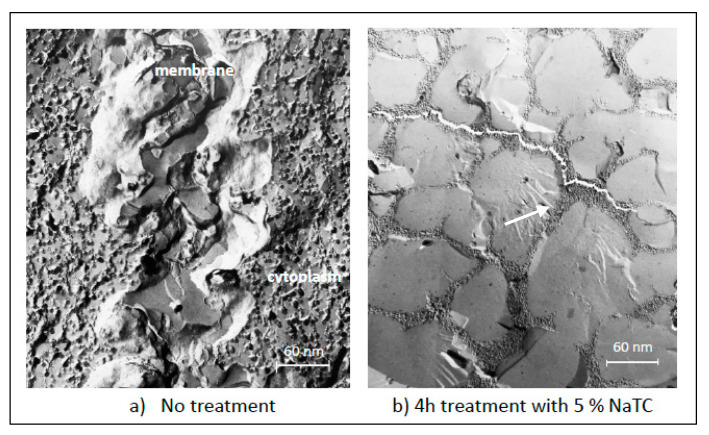
Freeze-fracture micrographs of porcine buccal mucosa: (**a**) intact and (**b**) treated for 4 h with sodium taurocholate (NaTC). The arrow depicts the lamellar and rough domains in cytoplasm and the absence of a membrane structure. The Methodology of Freeze-Fracture Microscopy for the micrographs given in Supplementary Materials.

There are two major routes involved in drug permeation across oral epithelium: the transcellular route (intracellularly—directly through the cells) and the paracellular route (intercellularly—through the spaces between the cells) [16]. In general, permeation across oral epithelial cells is largely achieved by simple passive diffusion and less by carrier-mediated transport. The permeability of the epithelium varies depending on the properties of a drug, such as lipophilicity, charge, molecular size. Small, lipophilic molecules can diffuse across the cell membrane, while intercellular permeation occurs with large hydrophilic molecules, such as peptides and proteins. TJs are the major selectively permeable barriers that control the paracellular transport of drugs. In order to enhance the permeation of the drugs across the oral mucosa, especially buccal and sublingual, various penetration enhancement strategies, such as chemical penetration enhancers, and physical methods, such as electric field and sonophoresis, have been investigated [17,18,19]. In early years, these studies were at a more basic level, but in recent years, there has been a transition to a more molecular level in regard to interactions with the junctional proteins. In an early study where the effect of bile salt as a penetration enhancer on buccal mucosa was investigated in vitro, upon 4 h of treatment with bile salts, significant penetration enhancement across porcine buccal mucosa was obtained for the FITC-dextrans (chosen as model compound for peptide drugs) at different molecular weights (4–10 kD) [20,21]. Furthermore, freeze fracture micrographs of the bile salt-treated epithelial cells were completely different than that of the intact epithelial cells, and instead of showing large membrane surfaces (Figure 3a), the fracture plane passed almost exclusively across the cytoplasmic space, showing icy and proteinaceous materials (Figure 3b). At that time, we had concluded that the bile salts most likely enhance the permeability of the mucosa by modifying the cell membrane integrity in such a way that the intracellular domain is opened up, and hence, the transepithelial pathway significantly shortened. Correlations between the flux data and histological observations were found to demonstrate that di- and trihydroxy bile salts behaved identically in both aspects. After showing that the bile salts enhance the buccal permeation of the drugs, in another study, we investigated the effect of bile salt (5% sodium glycodeoxycholate) on the permeation of a small, morphine sulfate across the buccal mucosa [22]. After 4 h of treatment with the bile salt, changes in epithelium at ultrastructural level, such as formation of vacuoles, swelling of the cells and possible increase in intercellular space, was observed. No tight junctions were observed anymore, while fewer desmosomes were observed. The effect of bile salt on epithelial barrier was also investigated using Fourier transform infrared spectroscopy (FTIR). Bile salt-treated tissue was compared to lipid-extracted (with solvents) tissue, and the results revealed an interaction between bile salts and the epithelial lipids. Presence of the bile salt was found to disrupt the epithelial lipids and, hence, to decrease the diffusional resistance to permeants. Furthermore, in another study, we have shown that there was a linear relationship between the accumulation of bile salt in the tissue and increase in penetration of the drug [23].

In the following years, different groups examined the relationship between the bile salts and tight junctions in more detail. Raimondi et al. [24] have demonstrated that treatment with deoxycholic acid, chenodeoxycholic acid but not ursodeoxycholic acid can induce epithelial growth factor receptor (EGFR) phosphorylation in Caco-2 monolayers, resulting in an increased paracellular permeability via occludin dephosphorylation and cytoskeletal rearrangement at the TJ level. Chen et al. [25] have also investigated the effect of bile acids on barrier function of squamous epithelial and tight junction (TJ) proteins. Upon treatment of cells with taurocholic acid, glycocholic acid or deoxycholic acid, disruption of the squamous epithelial barrier function was observed, which was attributed to modulation of claudin-1 and claudin-4. In another study, the effect of different enhancers, such as EDTA, sodium cholate, sodium dodecyl sulfate (SDS) and ethanol, on junction proteins was investigated [26]. It was shown that the enhancers at certain concentration and action time causes little cytotoxicity to Caco-2 cells and increased the permeability of FITC and FITC-dextrans, and produces changes in ZO-1, claudin-1, occludin and E-cadherin distribution. 

Various biomaterials such as chitosan have been shown the enhance the permeability of drugs across mucosa [27,28]. Different mechanisms have been suggested to explain the permeation enhancement of this polymer. Bioadhesive nature of chitosan, which increases the retention of the drug at its application site, as well as disruption of lipid organization of the cell membrane has been attributed to its penetration enhancing activity. Later, studies that describe this interaction in more detail were published. Yeh et al. [29] have investigated TJ disruption in the presence of chitosan at the gene and protein expression levels. Their data showed that chitosan exposure resulted in a significant increase in claudin-4 (CLDN4) gene transcription, which was observed to be reversible. Chitosan treatment induced redistribution of the TJ protein CLDN4 intracellularly, followed by its degradation in lysosomes, and resulted in opening of TJs. The recovery of TJ was observed after exposure to chitosan. It was concluded that chitosan regulates TJs by inducing changes in transmembrane CLDN4 protein. Smith et al. [30] have reported that chitosan-mediated TJ disruption was by translocation of TJ proteins (zona occludens 1 (ZO-1) and occludin) from the membrane to the cytoskeleton. Sonaje et al. [31] have also investigated the effects of chitosan on opening of TJs and paracellular transport at microscopic, ultrastructural and computed tomographic levels in Caco-2 cell monolayers and animal models. Chitosan treatment was observed to be associated with the translocation of JAM-1 (a trans-membrane TJ protein). It was demonstrated that chitosan treatment slightly opened the apical intercellular space and after removal of chitosan, the TJs were completely recovered. The authors have obtained similar microscopic and ultrastructural findings in in vivo studies performed in Wistar rats. 

The enhancers mentioned up to here are considered as non-specific TJ modulators. Nonetheless, recent studies have focused on specific modulation of the TJ-associated transmembrane proteins for enhancement of permeation of drugs across mucosa [32,33,34]. Specific peptide ligands of extracellular domains (ECDs) of TJ proteins have been designed, which affect single TJ proteins, allowing selective targeting of specific barriers, depending on the expression pattern of the TJ proteins. Furthermore, RNA interference techniques to downregulate the expression of targeted TJ proteins have been applied [35]. These TJ protein-targeted strategies have the potential to provide platforms for the development of novel therapies [36]. However, at present, application of these approaches for the oral mucosa remains very limited. Studies performed in human on oral mucosal delivery of drugs, especially for systemic effect, are generally based on formulations, which include non-specific enhancers such as surfactants, fatty acids, etc., as well as the mucoadhesive polymers, which also exert penetration enhancing property [37]. Nevertheless, for oral mucosal delivery, only the low-molecular-weight drugs successfully reached the marketplace, while there is still more to do for large molecules. The oral spray formulation of human insulin (Oral-Lyn), which consists of the combination of a surfactant, a solubilizer, a micelle creating agent and emulsifying agents, allowing insulin to permeate across the buccal mucosa can be given as an example for macromolecule delivery [38]; however, although approved in some countries, it does not have approval in North America or Europe yet. 

## 4. Oral Microbiota

The oral cavity has the second largest and diverse microbiota after the gut, harboring over 700 species of microbial communities, that has evolved to promote oral health and exists in a dynamic balance with the host [39]. In this habitat, microbes colonize the hard surfaces of the teeth and the soft tissues of the oral mucosa. Commensal microbiota is considered as the main driver of barrier immune function, shaping protective/homeostatic immune responses at the mucosal tissues. Yet, complex biofilms of varying compositions of bacteria can be formed, which are recognized as a virulence factor in many oral infectious diseases. These biofilms consist of complex microbial communities embedded in a matrix of polymers of bacterial and salivary origin [40]. Furthermore, some of these pathogens affect the epithelial barrier function by various mechanisms: they can manipulate the barrier related genes/proteins for their attachment and subsequent internalization, or directly destroy the junctions, thereby providing a gateway to the underlying tissue [41,42]. Recently, Takahashi et al. [43] have performed a search through an electronic database in regard to bacterial species, regulated barrier junction markers/genes and their mechanisms. They concluded that the periodontopathic bacteria contribute to epithelial barrier dysfunction in the gingiva by producing several virulence factors. However, some bacteria in the oral cavity were observed to be beneficial, helping gingival epithelial cells to maintain their integrity and barrier function. It was suggested that beneficial bacteria induce antimicrobial peptides (AMPs) through host immune response or express direct antimicrobial activity against barrier-disrupting pathogens. AMPs are considered to maintain the epithelial barrier by enhancing tight junction (TJ)-related gene expression. Nonetheless, the mechanism of these junctional modifications has not been fully elucidated and there are still controversies that need to be resolved. It is believed that there will not be a unified strategy that is employed by all pathogens. 

Microbiome is described as a community of commensal, symbiotic and pathogenic microorganisms within a body space or other environment [44]. Although the terms microbiome and microbiota are used interchangeably, in principle, the microbiota comprises all living members forming the microbiome [45,46]. The human microbiome is, indeed, an integral component in the maintenance of health. The Human Microbiome Project (HMP) has been running for over 10 years and in two phases to provide resources, methods and discoveries that link interactions between humans and their microbiomes to health-related outcomes [47,48,49]. As part of the HMP, the expanded Human Oral Microbiome Database (*e*HOMD) was created to provide the scientific community with comprehensive curated information on the bacterial species present in the human aerodigestive tract (ADT), which encompasses the upper digestive and upper respiratory tracts, including the oral cavity, pharynx, nasal passages, sinuses and esophagus [50]. 

The beneficial effects of the human microbiome depend on the balance within the microbiome and with the host [51]. Although the recent attention of the microbiome field has focused mostly on the gut, the oral microbiome is also vital in maintaining oral as well as systemic health. Furthermore, the oral cavity is the initiation point of digestion and a large number of oral microorganisms enter the digestive tract from the oral cavity through saliva, and they present a particularly close relationship with digestive diseases [52]. The oral cavity is exposed to many factors, such as pH changes in the oral cavity, diet of the host, nutrients, pharmacological factors and the external environment (e.g., climate and air pollution). All these factors play dominant roles in the modulation of the oral microbial community between different individuals. In the oral cavity, dental caries, periodontal disease and oral candidiasis are the major manifest diseases caused by an imbalance between the microbiota and the host (dysbiosis) [53,54,55]. Oral infections can lead to the extension of infection into surrounding tissues and to systemic infections. Dissemination of oral bacteria into the bloodstream (bacteremia) plays a role especially in infective endocarditis and prosthetic joint infection [56,57,58]. A good oral health status and satisfactory level of oral hygiene are considered to be sufficient to control the consequences of the systemic spread of oral microorganisms in healthy individuals. Furthermore, revised guidelines for the prevention of infective endocarditis published by the American Heart Association (AHA) in 2007 [59] recommend that caution is still needed and prophylactic antibiotics must be administered to susceptible or medically compromised patients, especially for all dental procedures that involve the manipulation of gingival tissue or the periapical region of teeth or perforation of the oral mucosa.

Furthermore, some specific bacterial strains have been recognized and strongly associated with other local diseases, such as oral cancer (*Capnocytophaga gingivalis*, *Fusobacterium* spp., *Streptococcus* spp., *Peptostreptococcus* spp., *Porphyromonas gingivalis* and *Prevotella* spp.) [60]. Hypotheses to explain how the oral microbiota is involved in cancer pathogenesis are mainly based on chronic inflammation, microbial synthesis of cancerogenic substances and alteration of epithelial barrier integrity. The interaction between oral epithelial cells and microbes provides oral cells with the capability of undergoing invasion and metastasis. This microbial interference has been reported to promote epithelial-mesenchymal transition (EMT), which allows polarized epithelial cells to mimic mesenchymal phenotype through various biochemical and molecular changes [61]. Microbial intervention has been shown to cause downregulation of important junctional proteins, such as E-cadherin and β-catenin, along with upregulation of mesenchymal markers, such as N-cadherin, vimentin and fibronectin. Hence, investigating the changes in these proteins has been suggested to be utilized as predictors for metastasis in squamous cell carcinoma of the oral cavity and oropharynx.

Dysbiosis is also associated with a range of systemic disorders, including inflammatory bowel diseases, autoimmune disease, obesity and metabolic syndrome, peripheral vascular disease and hypertension, aberrant responses to drugs, depression and autism [51]. Changes in the human microbiota may represent also an underlying factor of allergic diseases [62]. Commensal bacteria participate in the maintenance of immunological tolerance. Epithelial barrier dysfunction, particularly at the tight junction (TJ) has been reported to be essential for the pathogenesis of allergic diseases [63]. Disruption of epithelial barrier allows the penetration of allergens leading to allergic inflammation. Thus, asthma, rhinitis, chronic rhinosinusitis and allergic diseases have been demonstrated to be manifestations of a common systemic immune imbalance between the damaged-activated epithelium and immune cells. 

As a result, improving our knowledge of microbiomes and their interactions with physical and immunological barriers is important for diagnosis and treatment of numerous diseases. 

In the following sections, how specific microbes play crucial roles in tailoring immune cell functions in the oral cavity will be described after a brief introduction to the oral immune system.

## 5. Antimicrobial Peptides

Antimicrobial peptides (AMPs), also called host defense peptides, exerting a cationic nature, are part of the innate immune response. AMPs are considered as endogenously produced antibiotics, and they act at an early stage against microbial invasion [64]. Over 45 distinct antimicrobial peptides (AMPs) have been identified in human saliva and gingival crevicular fluid [65]. They are produced by the salivary glands and epithelial cells, and they form a continuous layer on the mucosal surfaces [66,67,68]. These AMPs have been reported to have distinct but overlapping roles in maintaining oral health and preventing bacterial, fungal and viral adherence and infection [68,69]. Defensins, cathelicidins (LL-37), calprotectins and histatins are the major AMPs detected in the oral cavity [70]. AMPs participate in a preservative co-evolution with the microbiome, and they help to maintain a balanced microbiota. Furthermore, apart from their antimicrobial activity, the AMPs have been reported to participate in several other crucial roles in host tissues, such as wound healing and cell proliferation, chemotactic for immune cells [71]. Recently, Salem et al. [72] have shown the involvement of hBD-2 in the pathogenesis of oral lichen planus (OLP), which is a common chronic mucocutaneous disorder with an immune mediated pathogenesis, and suggested that this AMP could be combined with therapeutic interventions in OLP. 

The role of the AMPs is just beginning to be understood, with potential applications for enhanced natural expression or as new therapeutic agents. In recent years, especially, the interplay between the antimicrobial peptides and gut microbiota has been the main focus of the studies in order to find new therapeutic strategies against various gut infections [73,74].

## 6. Oral Immune System

**Figure 4:**
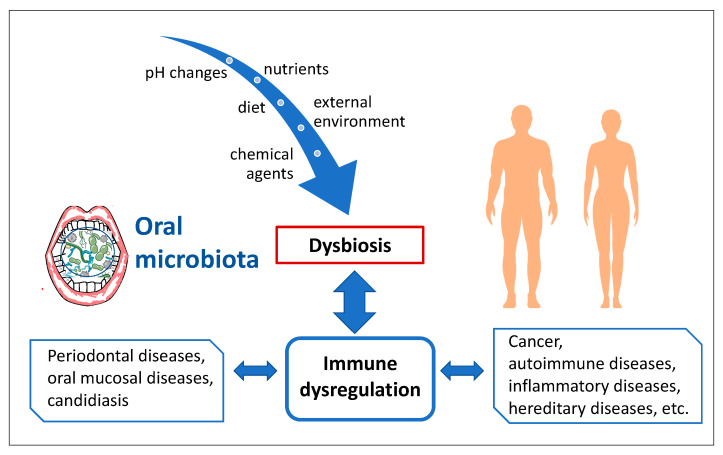
Relation between the immune system and microbiota in the oral cavity.

The mucosal immune system of the oral cavity is constantly exposed to tissue-specific signals, including a rich community of commensal microbes and their metabolites, continuous tissue damage from mastication, antigens from food and airborne particles, which present potential challenges to the homeostasis of the oral mucosa [53,75]. Hence, the oral mucosa and inherent mucosal immune system becomes very crucial for the protection of the integrity of the internal environment. Communication between the epithelium and innate and adaptive immune cells is fundamental for rapid recognition and effective elimination of pathogens at the epithelial surface. Both the host immune system and commensal microbiota can influence one another to maintain homeostasis. A dysbiosis in the microbiota leads to a dysregulation of the local immune response at that site (Figure 4).

The oral immune system is composed of three major compartments: the epithelial layer, *lamina propria* and the mucosal-associated lymphoid tissue (MALT). The cells of the innate immune system are located strategically at the host–microbiome interface. These cells have the ability to sense microorganisms or their metabolic products, and they translate the signals into host physiological responses to regulate the microbial ecology [76]. The pattern recognition receptors (PRRs) which are predominantly expressed on immune cells, identify the pathogen-associated molecular patterns (PAMPs) or molecules released by damaged cells (damage-associated molecular patterns-DAMPs). The currently identified PRR families are the Toll-like receptors (TLRs), C-type lectin receptors (CLRs), nucleotide-binding oligomerization domain-like receptors (NLRs), retinoic acid-inducible gene-I-like receptors (RLRs), and AIM2-like receptor (ALR). Upon PAMP recognition, PRRs, present at the cell surface or intracellularly, signal to the host the presence of infection and trigger proinflammatory and antimicrobial responses by activating a multitude of intracellular signaling pathways. PRR-induced signal transduction pathways ultimately result in the activation of gene expression and synthesis cytokines, chemokines, cell adhesion molecules and immunoreceptors, which together coordinate the early host response to infection and at the same time represent an important link to the adaptive immune response [77]. Innate immunity plays a unique role in oral immunity, by triggering a crucial systemic response to protect the host and maintain homeostasis. Furthermore, the innate defense is pivotal in the activation and regulation of adaptive immunity. The cytokine interleukin (IL-7)-mediated immune pathway is induced within hours following epithelial cell injury or activation of PRRs [78]. Despite the fact that IL-17 is described as a T cell-secreted cytokine, much of the IL-17 released during an inflammatory response has been shown to be produced by the innate immune cells. The innate immune cell populations that are an early source of IL-17 in response to stress, injury or pathogens are thought to reside in barrier tissues at the interface of host and environment. The epithelial cells are thought to exert manifold sentinel functions in perceiving pathogens and orchestrating the defense against them, besides their role as a physical barrier [79].

Additionally, fibroblasts, endothelial cells, chondrocytes and adipocytes respond to IL-17A by expressing antimicrobial proteins and peptides, and the proinflammatory cytokines and chemokines are involved in acute-phase responses and tissue remodeling [80]. IL-17 alone and in coordination with IL-22 was reported to induce the production of b-defensins (HBD), regenerating (ReG) proteins, S100 proteins and cathelicidins [81]. IL-17 also promotes epithelial cell secretion of chemokines for immune cell recruitment when the mucosal barrier is disrupted. IL-17A is recognized as the master regulator of host–microbiota interactions both in physiologic conditions and in immune-mediated inflammatory diseases [82,83,84]. 

Moutsopoulos and his group have comprehensively investigated the role of the microbiota and its metabolites in induction of the local immune cells of the oral mucosal immune system, focusing on the interleukin-17 (IL-17)/T helper 17 (TH17)-dependent pathways that control the two major oral infectious diseases, periodontitis and candidiasis [85,86]. They have demonstrated in animal and human studies that the microbiota-triggered Th17 cells are the drivers of local immunopathology and therapeutic targets in oral mucosal diseases. Furthermore, several groups have shown that the medication-related osteonecrosis of the jaw (MRONJ), which is a side effect of bisphosphonate therapy, is also associated with disfunction of the immune system, coupled with bacterial infection [87]. As a result, the oral epithelium, which acts as a physical barrier can no longer maintain its role. Hence, several strategies are being investigated to protect the oral mucosa from bisphosphonate exposure [88,89]. Likewise, immunological changes occurring in pregnancy may also induce a dysbiosis of the oral microbiota and contribute to increased inflammation of periodontal tissues. Pregnant women with periodontal disease have been reported to be at increased risk of adverse pregnancy outcomes, including preeclampsia, preterm delivery and low birth weight [90,91]. However, how periodontitis may lead to adverse pregnancy outcomes is not yet fully understood.

On the other hand, despite high bacterial colonization and frequent allergen contact, acute inflammatory and allergic reactions are rarely seen in the oral mucosa [92]. It is suggested that a very potent immune tolerance predominates this site to monitor and control the interactions between host innate defense and microbiota [93]. 

## 7. Crosstalk between Immune System and Microbiota

As a consequence of the basic symbiotic way of life, microbiota and innate immunity engage in an extensive bidirectional communication. While the immune system affects and preserves the microenvironment for the microbiota, the host microbiota re-adjusts and promotes the immune system to be tolerant toward the commensal and useful members of the microbiota [94]. Both the microbiota and immune system also communicate for necessary responses against the pathologic and harmful microorganisms. A very finely regulated and highly coordinated interaction between the immune system and microbiota is required to achieve both local and systemic homeostasis while preserving host biological integrity. In this direction, symbiosis and dysbiosis are the two extremes of the complex relationships between the oral microbial community and the immune responses to its presence. Many multifactorial disorders are believed to be influenced and/or driven by alteration of the intimate crosstalk between immune system and the microbiota [76].

In recent years, studies on microbiota metabolites and their interactions with the immune system has also brought a new dimension to the understanding of host–microbiota interactions. It is suggested that sensing of bacterial metabolites by the host is much more informative about the state of microbial colonization than recognition of microbial surface molecules, as metabolites provide information about the activity and function of microorganisms, rather than their presence or absence [95].

## 8. Personalized Medicine

Recent advances in immunology, genetics, and microbiology have guided to a new era in the continued efforts to better understand and treat oral diseases, moving ever closer to predictive, preventive and personalized medicine (PPPM) [94,96]. PPPM is the new integrative concept in the health care sector that enables to predict the individual predisposition before onset of the disease, to provide targeted preventive measures and create personalized treatment algorithms tailored to the person, with the aim to curb the prevalence of both communicable and non-communicable diseases. In the era of personalized medicine, it is crucial that oral health is also integrated into this concept. Various approaches have been suggested about how knowledge of the oral microbiome may be utilized for personalized dentistry at the point of care [97]. Monitoring of microbiomic and metabolomic changes during the transition from oral health to disease becomes important in understanding which will help the healthcare to prevent the disease before it occurs. In this regard, application of the knowledge of the human microbiome should aim at preserving the highly intra- and inter-individual diversity of the oral microbiota, while protecting its loss. The application of personalized medicine in dentistry using a combination of microbiomeand genomic information includes periodontal diseases, caries, oral cancer, orofacial pain, etc. [98,99]. Personalized medicine has the potential to mitigate the chronic and often destructive nature of these disorders by taking a more proactive approach to disease diagnosis and therapy, rather than the currently applied reactive, wait-and-see approach [100]. It is certainly imperative for the oral health community to be aware of the opportunities and challenges in personalized medicine. 

## 9. Conclusions

In general, there is a close relationship between general health/disease and oral mucosal reactions. The oral mucosa functions as a barrier to protect the deeper tissues from mechanical insults and to prevent the entry of pathogens as well as the exogenous harmful substances in order to maintain the tissue integrity and homeostasis. It consists of physical (cell–cell and cell–extracellular matrix junctions, especially TJs), microbiological and immune barriers. Understanding the functions of these barriers and interactions between them in more detail has become vital for diagnosis, prevention and therapy of a plethora of local and systemic diseases, including inflammatory and infectious, cancer, cardiovascular, diabetes, etc. The microenvironment of oral barriers harbors over 700 species of microorganisms, which are regulated through sophisticated signaling systems and driven by host and environmental factors. Recent findings in immune system and microbiome provides a new perspective to this field, enhancing our understanding of the intimate but complicated crosstalk between the microbiome and the immune system. The reciprocal interactions between the microbiota and immune system shape the mucosal homeostasis or dysbiosis and ultimately health or disease. Furthermore, similar communal alterations have also been demonstrated for the physical barriers in relation with microbiota-immune system interaction. Therefore, it is important to consider the relations between the physical, microbiological and immune barriers as a whole in order to understand the underlying reason of a disorder or disease, for successful prophylactic and therapeutic approaches. While there are still many unknowns and challenges in the elucidation of the interactions in homeostasis and disease, rapid advances in this area are, indeed, very promising and the new findings certainly provide new insights for future development of diagnostics and therapeutics for personalized medicine as well as provision of prevention of the disease.

## Supplementary Materials

Methodology of Freeze-Fracture Microscopy for the micrographs given in Figure 2 and Figure 3 are available online at https://www.mdpi.com/article/10.3390/ijms22157821/s1.
