# Supplementary material for: An Overview of Physical, Microbiological and Immune Barriers of Oral Mucosa
Source: Int J Mol Sci. 2021 Jul 22;22(15):7821. doi: 10.3390/ijms22157821 (PMC8346143; doi:10.3390/ijms22157821)
Supplement: Supplementary file 1 [file ijms-22-07821-s001.zip › ijms-1301462-SI.pdf]

Methodology of Freeze-Fracture Microscopy for Figures 2 and 3

Porcine buccal mucosa, which satisfactorily mimics the non-keratinized human buccal mucosa was freshly obtained from a local slaughterhouse and was used within 2h upon removal. Most of the underlying tissue was removed from the mucosa with surgical scissors, then the epithelium was separated from the remaining connective tissue with an electro-dermatome. Tissue samples (1 X 8 mm) were folded into small cylinders and cryofixed by rapid freezing (10^5K/sec) in liquid propane using the plunging method (KF80 ReichertJung, Austria). The frozen samples were fractured in a freeze-etching device (Bakers BAF400D) at a sample temperature of - 150°C and at a pressure of 10^{-6} Torr. Replication was performed by platinum/carbon evaporation (2.5 nm) at an angle of 45° and additional carbon evaporation (35 nm) perpendicular to the fracture plane. After replication, the specimens were removed from the vacuum chamber, thawed and submerged in 0.5 M undecyl-dodecyl-dimethyl ammonium hydroxide in toluene (Soluene-350), Fresh soluene was replaced every 24 h for 6 days. Finally, the replicas were washed with toluene, dichromate sulphuric acid and water, respectively. Replicas were collected onto 400 mesh copper grids and examined in the transmission electron microscope (Philips EM201C).
